# Editorial: Intersection of biophysical and structural approaches in vaccine design

**DOI:** 10.3389/fmolb.2023.1142656

**Published:** 2023-02-14

**Authors:** Christopher P. Ptak, Ching-Lin Hsieh, James A. Garnett, Rino Rappuoli, Yi-Pin Lin

**Affiliations:** ^1^ NMR Facility, Carver College of Medicine, University of Iowa, Iowa City, IA, United States; ^2^ Department of Molecular Biosciences, The University of Texas at Austin, Austin, TX, United States; ^3^ Sanofi S.A., Cambridge, MA, United States; ^4^ Centre for Host-Microbiome Interactions, Faculty of Dental, Oral and Craniofacial Sciences, King’s College London, London, United Kingdom; ^5^ Monoclonal Antibody Discovery (MAD) Lab, Fondazione Toscana Life Sciences, Siena, Italy; ^6^ Department of Biotechnology, Chemistry and Pharmacy, University of Siena, Siena, Italy; ^7^ GSK, Siena, Italy; ^8^ Division of Infectious Diseases, Wadsworth Center, New York State Department of Health, Albany, NY, United States; ^9^ Department of Biomedical Sciences, SUNY Albany, Albany, NY, United States

**Keywords:** vaccine design, antigen, protein engineering, stability, structural biology

Infectious diseases have always been a major threat to the prosperity of society. The COVID-19 pandemic is evidence that contagious diseases are evolving to remain as relevant as ever in our modern interconnected world. In recent years, vaccine development has been a bright spot and entered a golden age that was ushered in with help from the intimate knowledge of molecules tied to the disease-causing pathogens (Rappuoli et al., 2016; Graham et al., 2019; Rappuoli, 2021). Utilization of the structure and biophysical properties of antigens has proven to be one of the most productive applications of structural biology promising to continue to save lives into the future. This Research Topic highlights studies that build molecular insights into the antigenic targets involved in several important diseases.

Bacterial pathogens with resistance to traditional antibiotics pose a serious health risk and are therefore the target of numerous vaccine development efforts. Some of the first pioneering work establishing structure-based vaccines as an effective strategy were developed against *Neisseria* -linked bacterial meningitis (Malito et al., 2014). The article by Pietri et al. examines the potential for polysaccharide-based vaccines in combating an emerging *Neisseria meningitidis* serogroup. The study examines monoclonal antibody-immunogenic epitope interactions with a combination multiple biophysical and structural techniques, including ELISA, SPR, NMR, X-ray crystallography, and *in silico* docking analysis. The authors identified a minimal polysaccharide length required for immunogenic response. *Mycobacterium tuberculosis*, the causative agent of tuberculosis, is another well-known and dangerous pathogenic bacterium with significant global interest in its eradication. In the article by Ruggiero et al., a structural model of the protein antigen, HtpG_Mtb_, was supported by evidence from biophysical experiments and utilized to predict the region of the protein with immunogenic epitopes. A rationally designed HtpG_Mtb_-derived antigen of optimal length and stability was experimentally determined to have high immunoreactivity. These articles illustrate the advantage of merging insights from multiple structural and biophysical approaches to aid in the engineering of vaccine antigens.

Vaccine development in viruses has advanced more rapidly as the generally smaller genome size offers fewer targets to dissect; however, the multifunctional and dynamic nature of viral proteins can make their study challenging. The sudden widespread emergence of SARS-CoV-2 in 2020 was met with the rapid development of effective vaccines and demonstrated a powerful role for structural and biophysical characterization (Hsieh and McLellan, 2022). In a computational study, Masoomi Nomandan et al. examined the effects of glycosylation on a nanoparticle composed of the SARS-CoV-2 receptor binding domain and ferritin. Molecular dynamics simulations revealed an enhancement in nanoparticle stability when glycosylation was positioned along protein-protein interfaces. Beeckmans et al. provides an informative review of several SARS-CoV-2 structural proteins of interest to vaccine development. The authors describe bioinformatic tools that are easily accessible to help the average biochemist, immunologist, or infectious disease specialist build a detailed molecular understanding of the key features of antigenic targets. The lessons-learned from SARS-CoV-2 vaccine research has provided a blueprint for vaccine development against other serious viruses illustrating how to effectively apply structural and biophysical knowledge to the process.

The aim of this Research Topic is to highlight biophysical and biochemical studies and methods that bridge the gap between the molecular-level details of a native antigen afforded by a structure and the final engineered vaccine. This topic is composed of articles that explore the structural and biophysical properties of antigenic molecules and assemblies thereby providing knowledge that may be useful in vaccine development. Continued work in the field of structure-based vaccine design is critical to maintain a broad knowledge base of our microscopic enemies’ armor, camouflage, and artillery as well as their strategies. The editors are grateful for the contribution of the authors and reviewers to this Research Topic as well as to the field of vaccine research.
